# Supplementary material for: Socio-economic factors associated with cancer stigma among apparently healthy women in two selected municipalities Nepal
Source: PLoS One. 2024 Dec 16;19(12):e0301059. doi: 10.1371/journal.pone.0301059 (PMC11649127; doi:10.1371/journal.pone.0301059)
Supplement: S1 Dataset — (DOCX) [file pone.0301059.s001.docx]

Cancer Stigma and cervical cancer screening attendance: a population based study in Nepal Questionnaire

Participants ID number Name of the interviewer

# Eligibility Questions

1. How old are you? Has to be between 30 to 60 years.
2. Is you resident of Dhulikhel or Banepa?

**Consent:** Agree Disagree

# Part 1: Socio-demographic and reproductive health Socio-demographic characteristics

1. Ethnic Group
   - Brahmin
   - Chettri/Thakuri/Sanyasi
   - Newar
   - Magar/Tamang/Rai/Limbu
   - Sherpa/Bhote
   - Kami/Damai/Sarki/Gaaine/Baadi
   - Others If others specify
2. Marital Status
   - Married
   - Unmarried
   - Separated
   - widowed
   - Cohabiting
   - Refused
3. Religion
   - Hindu
   - Buddhist
   - Muslim
   - Kirat
   - Christian
   - Others---specify
4. What is the highest grade or year of school you have ever completed, including college
5. Which of the following describes your main status over the past 12 months?
   - Government Employ
   - Non-Government Employ
   - Self Employed
   - Non Paid
   - Home maker
   - Unemployed
   - Student
   - Farmer
   - Teacher
   - Health worker
   - Business
   - Others If others specify
6. Talking about the past year, what was your average earning?
7. Talking about the past year, what was your family’s annual earning?
8. How many members are there in your family (share kitchen)?
9. How old was your husband on his last birthday?
10. What is the highest grade your husband completed?
11. What is your husband’s occupation? That is. What kind of work does he mainly do?

- Government Employ
- Non-Government Employ
- Self Employed
- Non Paid
- Home maker
- Unemployed
- Student
- Farmer
- Teacher
- Health worker
- Business
- Others

1. Do you currently smoke?
   - Yes
   - No
2. Did you ever smoked in the past?
   - Yes
   - No
3. Do you currently consume any form of alcohol?
   - Yes
   - No
4. Did you ever consumed any form of alcohol?
   - Yes
   - No

# Reproductive health information

1. Have you ever given birth?
   - Yes
   - No
   - Refused
2. How many children that you gave birth?

# Part 2: Cancer stigma scale

| SN |  | Disagree strongly | Disagree moderately | Disagree slightly | Agree slightly | Agree moderately | Agree strongly |
| --- | --- | --- | --- | --- | --- | --- | --- |
| Awkwardness | | | | | | | |
| 1 | I would feel at ease around someone with  cancer(R) |  |  |  |  |  |  |
| 2 | I would feel comfortable  around someone with cancer (R) |  |  |  |  |  |  |
| 3 | I would find it difficult being around someone with cancer |  |  |  |  |  |  |
| 4 | I would find it hard to talk to someone with cancer |  |  |  |  |  |  |
| 5 | I would feel embarrassed  discussing cancer with someone who had it |  |  |  |  |  |  |
| Severity | | | | | | | |
| 6 | Once you’ve had cancer  you’re never ‘normal’ again |  |  |  |  |  |  |
| 7 | Having cancer usually ruins a person’s Career |  |  |  |  |  |  |
| 8 | Getting cancer means  having to mentally prepare oneself for death |  |  |  |  |  |  |
| 9 | Cancer usually ruins close personal relationships |  |  |  |  |  |  |
| 10 | Cancer devastates the lives of those it touches |  |  |  |  |  |  |
| Avoidance | | | | | | | |
| 11 | If a colleague had cancer I would try to avoid them |  |  |  |  |  |  |
| 12 | I would distance myself  physically from someone with cancer |  |  |  |  |  |  |
| 13 | I would feel irritated by someone with cancer |  |  |  |  |  |  |
| 14 | I would feel angered by someone with cancer |  |  |  |  |  |  |

| 15 | I would try to avoid a person with cancer |  |  |  |  |  |  |
| --- | --- | --- | --- | --- | --- | --- | --- |
| Policy Opposition | | | | | | | |
| 16 | More government funding should be spent on the  care and treatment of those with cancer (R) |  |  |  |  |  |  |
| 17 | The needs of people with  cancer should be given top priority (R) |  |  |  |  |  |  |
| 18 | We have a responsibility to provide the best possible care for people with cancer (R) |  |  |  |  |  |  |
| Personal Responsibility | | | | | | | |
| 19 | A person with cancer is liable for their condition |  |  |  |  |  |  |
| 20 | A person with cancer is  accountable for their condition |  |  |  |  |  |  |
| 21 | If a person has cancer it’s probably their fault |  |  |  |  |  |  |
| 22 | A person with cancer is to blame for their condition |  |  |  |  |  |  |
| Financial discrimination | | | | | | | |
| 23 | It is acceptable for banks to refuse to make loans to people with cancer |  |  |  |  |  |  |
| 24 | Banks should be allowed to refuse mortgage  applications for cancer- related reasons |  |  |  |  |  |  |
| 25 | It is acceptable for insurance companies to  reconsider a policy if someone has cancer |  |  |  |  |  |  |
|  | R = Item reversed during calculating mean scores | | | | | | |

**Thank you**
