# Supplementary material for: Socio-economic factors associated with cancer stigma among apparently healthy women in two selected municipalities Nepal
Source: PLoS One. 2024 Dec 16;19(12):e0301059. doi: 10.1371/journal.pone.0301059 (PMC11649127; doi:10.1371/journal.pone.0301059)
Supplement: S1 File — As a part of the co-submission. (PDF) [file pone.0301059.s003.pdf]

RESEARCH ARTICLE

Association between cancer stigma and cervical cancer screening uptake among women of Dhulikhel and Banepa, Nepal

Bandana Paneru[1,2]*, Aerona Karmacharya[1], Alina Bharati[1], Soniya Makaju[1], Bikram Adhikari[1], Dikshya Kafle[3], Sunila Shakya[4], Donna Spiegelman[5,6], Sangini Seth[7], Anne Stangl[8], Aamod Dhoj Shrestha[9,10], Archana Shrestha[1,2,5]

1 Department of Public Health and Community Programs, Kathmandu University School of Medical Sciences, Dhulikhel, Nepal, **2** Institute for Implementation Science and Health, Kathmandu, Nepal, **3** Canadian Red Cross, Country Office Nepal, Kathmandu, Nepal, **4** Department of Obstetrics and Gynecology, Dhulikhel Hospital/Kathmandu University School of Medical Sciences, Dhulikhel, Nepal, **5** Center of Methods for Implementation and Prevention Science, Yale School of Public Health, New Haven, CT, United States of America, **6** Department of Biostatistics, Yale School of Public Health, New Haven, CT, United States of America, **7** Department of Obstetrics, Gynecology, and Reproductive Sciences, Yale School of Medicine, New Haven, CT, United States of America, **8** International Center for Research on Women, Washington, DC, United States of America, **9** Center for Global Health, Department of Public Health, Aarhus University, Aarhus, Denmark, **10** COBIN, Nepal Development Society, Bharatpur, Nepal

* bandana.paneru@gmail.com

OPEN ACCESS

Citation: Paneru B, Karmacharya A, Bharati A, Makaju S, Adhikari B, Kafle D, et al. (2023) Association between cancer stigma and cervical cancer screening uptake among women of Dhulikhel and Banepa, Nepal. PLoS ONE 18(5): e0285771. https://doi.org/10.1371/journal.pone.0285771

Editor: Cecilia Acuti Martellucci, University of Ferrara: Universita degli Studi di Ferrara, ITALY

Received: September 28, 2022

Accepted: April 29, 2023

Published: May 18, 2023

Copyright: © 2023 Paneru et al. This is an open access article distributed under the terms of the Creative Commons Attribution License, which permits unrestricted use, distribution, and reproduction in any medium, provided the original author and source are credited.

Data Availability Statement: All relevant data are within the manuscript and its Supporting Information files.

Funding: This study is funded by National Cancer Institute. The grant number is P30CA016359 (08/2020 – 07/2022.

Competing interests: The authors have declared that no competing interests exist.

Abstract

Background

Cervical cancer ranks as the most common cancer among Nepalese women with a high incidence and mortality. Despite evidence that effective screening programs reduce disease burden, screening services are under-utilized. Cancer stigma can be a major barrier to cervical cancer screening uptake among Nepalese women.

Objectives

This study assessed the association between cancer stigma and cervical cancer screening uptake among women residing in semi-urban areas of Kavrepalanchok district (Dhulikhel and Banepa), Nepal.

Methods

We conducted a cross-sectional study among 426 women aged 30–60 years using telephone interview method from 15th June to 15th October 2021. A validated Cancer Stigma Scale (CASS) was used to measure cancer stigma and categorized women as presence of cancer stigma if the mean total score was greater than three. We obtained information on cervical cancer screening uptake through self-reported responses. Univariable and multivariable logistic regression were performed to assess the association between cancer stigma and cervical cancer screening uptake. We adjusted socio-demographic: age, ethnicity, occupation, religion and education, and reproductive health variables: parity, family planning

user, age of menarche and age at first sexual intercourse during multivariable logistic regression.

Results

Twenty-three percent of women had cancer stigma and 27 percent reported that they had ever been screened for cervical cancer. The odds of being screened was 0.23 times lower among women who had stigma compared to those who had no stigma (95% CI: 0.11–0.49) after adjusting for confounders: age, ethnicity, occupation, religion, education, parity, contraceptive use, age of menarche and age at first sexual intercourse.

Conclusion

Women residing in semi-urban areas of Nepal and had cancer stigma were less likely to have been screened for cervical cancer. De-stigmatizing interventions may alleviate cancer stigma and contribute to higher uptake of cervical cancer screening.

Introduction

Globally, cervical cancer is the fourth most common cancer among women with an estimated 604,000 new cases and 342,000 deaths annually in 2020 [1]. Around 90 percent of deaths due to cervical cancer occur in Low and Low Middle Income Countries (LMIC) [2]. In Nepal, cervical cancer is the most common cancer, about 1,493 deaths occur annually in 2020 [3]. Cervical cancer is preventable with good-quality screening and vaccination against human papillomavirus (HPV), the cause of almost all cervical cancer cases [4]. The widespread use of screening has resulted in a steep decline in cervical cancer mortality in high-income countries [5].

In 2010, the Government of Nepal (GoN) developed national guidelines for Cervical Cancer Screening and Prevention (CCSP) with the goal of screening at least 50% of the target population, women aged 30–60 years. GoN provides free cervical screening in primary health centers through Visual Inspection with Acetic acid (VIA) approach and recommends screening every 5 years [6]. In our study sites, VIA is offered free of cost from government health facilities. Liquid based cytology(LBC) and HPV testing is available in community based hospitals that costs around NPR. 1200 for LBC and NPR 2500 for HPV testing.

Despite the improvements in treatment and survival, cervical cancer is still a stigmatized disease, characterized by exclusion, rejection, blame, or devaluation resulting from an adverse social judgment about the patient [7]. The experience of cancer stigma related to shame and blame appeared highest among patients with cervical cancer as it is linked with sexually transmitted infections [8, 9]. Cancer stigma and discrimination is related to being labeled based on physical appearance of perceived signs of cancer [10]. Furthermore, the huge cost of cancer treatment linked with poor prognosis lead to a stigma of draining family resources [11, 12]. These stigma negatively impact both patients diagnosed with cancer and the broader community by creating adverse psychosocial and health outcomes, poor quality of life for cancer patients, delays in seeking care, prognosis or treatment, work place discrimination, reduced availability of health services, and discriminatory behavior from health care providers, friends and relatives [13].

Cancer stigma is negatively associated with cervical cancer screening uptake around the world [14–17]. Psychological and emotional barriers like shyness, embarrassment,

defenselessness, and discomfort with exposing their body and fear of the result of the test has prevented women from participating in cervical cancer screening. In addition, women consider screening tests useless because of the belief that cervical cancer is unpreventable and incurable and has a high economic burden [11, 14, 18]. In Nepal, only 8 percent of the women aged 30–49 years were ever screened for cervical cancer in 2019 [19]. Qualitative studies have identified stigma as a potential barrier to cervical cancer screening uptake in Nepal but has not assessed association of cancer stigma with cervical cancer screening uptake quantitatively [20]. Studies on cervical cancer screening uptake and cancer stigma are very limited in LMICs like Nepal where the disease burden is on the rise. To our knowledge this is the first study to determine the association between cancer stigma(domains:awkwardness, severity, avoidance, policy opposition, financial discrimination and personal responsibility) and cervical cancer screening uptake globally.

This study assessed the association between cancer stigma and cervical cancer screening uptake among women of semi-urban areas in central Nepal.

Methods

Study design and settings

We conducted a cross-sectional survey of women aged 30–60 years from Dhulikhel and Banepa, Nepal. Dhulikhel and Banepa are ancient cities of Kavrepalanchok district, located about 30 kilometers east of the capital city (Kathmandu) with a total population of 39, 047 [21].

Participants

We collected data from a convenience sample of 426 women, aged 30–60 years residing in Dhulikhel or Banepa municipality. Women aged 30–60 years were selected based on the CC screening target group mentioned by national guidelines for Cervical Cancer Screening and Prevention. Those women with hearing impairment and mental disorders were excluded. The sample size was estimated with 80% power and 95% confidence interval to detect 46% screening among non-stigmatized and 31% screening among stigmatized women, adjusting for 10 percent non-response [22, 23].

We received the list of 30 to 60 years old women from Female Community Health Volunteers(FCHVs). FCHVs are front line pillars of community-based health programs in Nepal. They visit every household and advocate healthy behaviour by mothers and community people to promote safe motherhood, child health, and family planning and other community based health issues and service delivery. Female community health volunteers and social mobilizers in the study area identified and connected the potentially eligible participants to the research team. Research team contacted participants through telephone and provided study information. Verbal informed consent was obtained from participants and audio recorded with their consent. This study was approved by Kathmandu University Institutional Review Committee (KUIRC no: 35/2021; 9th May 2021).

Data collection

Trained research assistants interviewed the participants in Nepali by telephone from 15th June to 15th October 2021, using a structured questionnaire and entered responses into an electronic database using Kobotool.

Measures

Socio-demographic and reproductive health variables. Socio demographic variables included age (in years), ethnicity (Brahmin, Chettri/Thakuri/Sanyasi, Newar, Magar/Tamang/Rai/Limbu, Sherpa/Bhote, Kami/Damai/Sarki/Gaaine/Baadi, Other), education (number of years of formal education completed), religion (Hindu, Buddhist, Christian), occupation (home-maker, farmer, business, unemployed, others). Reproductive health information included parity (number of children), current contraception use(yes,no), age of menarche (in years) and age at first sexual intercourse (in years). The questions were adopted from previously conducted national surveys of Nepal [24, 25].

Cancer stigma. We measured cancer stigma using the Cancer Stigma Scale (CASS) [26] in Nepali language. We translated the CASS tool into Nepali language and was back translated by independent researchers. We pretested the tool among 30 participants and calculated cronbach alpha for scale and domains (scale- 0.81, awkwardness- 0.80, severity- 0.79, avoidance- 0.73, policy opposition- 0.81, personal responsibility-0.80 and financial discrimination- 0.83).

CASS has 25 items assessing six domains: (a) awkwardness: items measured how much people feel comfortable around someone with cancer, (b) severity: items measure how severe the consequences of a cancer diagnosis are expected to be and the likelihood of recovery from cancer, (c)avoidance: assess how much people avoid cancer patient and maintain physical distance with them, (d) personal responsibility: determine how a person's actions are considered to

Table 1. Socio-demographic characteristics of participants (n = 426).

Characteristics	Frequency (%)
**Age(years),** Mean(SD)	42.3 (8.1)
**Ethnicity**	
Brahmin/Chettri/Thakuri/Sanyasi	184 (43.2)
Newar	175 (41.1)
Magar/Tamang/Rai/Limbu	15 (3.5)
Sherpa/Bhote	27 (6.3)
Kami/Damai/Sarki/Gaaine/Baadi	25 (5.9)
**Religion**	
Hindu	374 (87.8)
Buddhist	28 (6.6)
Christian	24 (5.6)
**Educational status**	
No formal education	132 (31.0)
Primary	49 (11.5)
Secondary	150 (35.2)
Above secondary	95 (22.3)
**Occupation**	
Farmer	168 (39.5)
Homemaker	106 (24.9)
Business	63 (14.8)
Unemployed	7 (1.6)
Others	82 (19.2)
**Parity (number)**,Mean(SD)	2.3 ± 1.1
**Current contraceptive users**	223(52.3)
**age of menarche(years)**, Mean(SD)	14.1± 1.7
**age at first sexual intercourse (years)**,Mean(SD)	19.5 ± 3.8

https://doi.org/10.1371/journal.pone.0285771.t001

have contributed to their cancer, (e) policy opposition: items assess how much government and public are responsible towards care and treatment of cancer patients and (f) financial discrimination: measure how much cancer patients are expected to be benefited from bank and insurance services. The participants' responses were recorded on a 6-point Likert scale ('disagree strongly' to 'agree strongly), higher score indicating higher stigma [14]. We calculated mean scores for each of the domains after reversing the score of 5 items that indicated positive statements from the domains policy opposition and awkwardness [14, 26, 27]. The mean score was dichotomized into (a) no stigma (score 1 to 3) and stigma (3 to 6) [14].

Cervical cancer screening uptake. We assessed cervical cancer screening uptake from the self-reported responses to the question–"Has a health-care worker ever tested you for cervical cancer?"

Data analysis

Categorical data were reported in frequency and percentage; and numerical data with means and standard deviation. Cancer stigma prevalence was calculated on six domains as mentioned earlier. Clopper-Pearson method was used to determine the confidence interval for cancer stigma prevalence [28]. We used univariable and multivariable logistic regression models to assess the association between cancer stigma and cervical cancer screening uptake. In the multivariable model, we adjusted for socio-demographic variables (age in years, ethnicity, occupation education, religion) and reproductive health variables (parity, age of menarche, family planning current user and age at first sexual intercourse) based on prior literature review. We reported crude and adjusted odds ratio with 95% confidence interval and p-value. All analyses were conducted using STATA version 13.0 (Stata Corp., College Station, Texas, USA) for cleaning, coding and statistical analysis.

Table 2. Screening behavior of participants (n = 426).

Characteristics	Frequency (%)
**Ever Screened**	113 (26.5)
**Time since the last screening**	
< 1 year ago	33 (29.2)
1–2 years ago	33 (29.2)
3–5 years ago	21 (18.6)
>5 years ago	26 (23.0)
**Method of the last screening**	
HPV testing	3 (2.6)
VIA testing	2 (1.8)
Pap smear	28 (24.8)
Don't know	80 (70.8)
**Reasons for screening (n = 113)**	
Experienced symptoms	7 (6.2)
Part of routine examination	44 (38.9)
Recommended by health provider	21 (18.6)
Recommended by others	23 (20.3)
Others	18 (16.0)

https://doi.org/10.1371/journal.pone.0285771.t002

Results

Socio-demographic characteristics of the participants are summarized in Table 1. Participants' age ranged from 30 to 60 years with the mean of 42.3 ± 8.1 years. Majority (43%) were Brahmin/Chhetri; about one-third (31%) women had no formal education and the majority (40%) were engaged in agriculture. More than half (52.3%) were current contraceptive users and mean age of first sexual intercourse was 19.5 ± 3.8 years.

Twenty-six percent of the participants reported ever having been screened for cervical cancer. Among the screened participants, about 23 percent reported not having screened in the past five years. The majority (71%) of respondents were unaware of the screening method (Table 2).

Twenty-three percent of the participants had cancer stigma. Seventy-six percent of them had stigma on personal responsibility i.e. they perceive that a cancer patient is responsible for the cause of cancer. More than half (55%) had severe stigma. i.e. they perceive that cancer is terminal disease and cancer patients cannot get back to their pre-cancer state (Fig 1).

Association between cancer stigma and cervical cancer screening

There was a significant negative association between cancer stigma and cervical cancer screening uptake ($p<0.001$). The odds of being screened was 77% lower among those who had stigma compared to those who had no stigma (95% CI: 0.11–0.49;) after adjusting for age, ethnicity, occupation, religion, parity, education, current contraceptive user, age at menarche and age at first sexual intercourse (Table 3).

Within six cancer stigma domains, odds of being screened was 71% lower among participants having awkwardness stigma (95% CI: 0.15–0.54; $p<0.001$); 47% lower among participants having severity stigma (95% CI: 0.33–0.86; $p = 0.01$); and 51% lower among participants with financial discrimination stigma (95% CI: 0.30–0.80; $p = 0.004$) in multivariable model. The odds of being screened was 2.06 times higher among participants having personal responsibility stigma (95% CI: 1.12–3.79; $p = 0.019$) (Table 3).

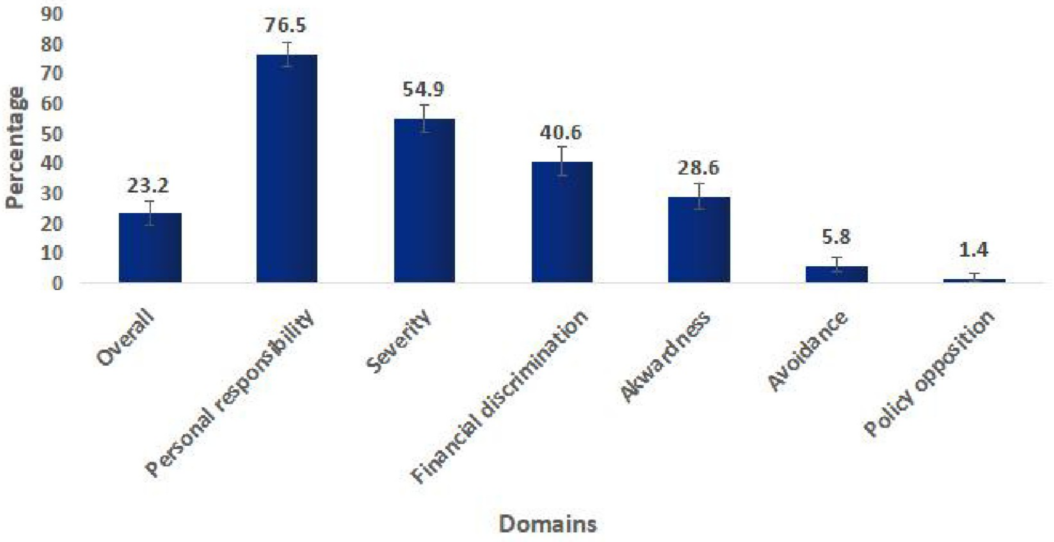

Fig 1. Prevalence of cancer stigma in six domains.

https://doi.org/10.1371/journal.pone.0285771.g001

Table 3. Association between cancer stigma and self-reported cervical cancer screening uptake (n = 426).

	Screening			Univariable			Multivariable	
	Yes n(%)	No n(%)	OR	95% CI	p-value	aOR*	95% CI	p-value
**Overall**								
No stigma	102(31.2)	225(68.8)	Ref					
Stigma	11(11.1)	88(88.9)	0.3	0.14,0.45	<0.001	0.23	0.11,0.49	<0.001
**Awkwardness**								
No stigma	98(32.2)	206(67.8)	Ref					
Stigma	15(12.3)	107(87.7)	0.3	0.16,0.53	<0.001	0.29	0.15,0.54	<0.001
**Severity**								
No stigma	61(31.8)	131(68.2)	Ref					
Stigma	52(22.2)	182(77.8)	0.6	0.39,0.94	0.027	0.53	0.33,0.86	0.01
**Avoidance**								
No stigma	109(27.2)	292(72.8)	Ref					
Stigma	4(16.0)	21(84.0)	0.5	0.17,1.52	0.228	0.65	0.20,2.04	0.459
**Policy Opposition**								
No stigma	113(26.9)	307(73.1)	Ref					
Stigma	0(0)	6(100%)	1			1		
**Personal responsibility**								
No stigma	18(18.0)	82(82.0)	Ref					
Stigma	95(29.0)	231(70.9)	1.9	1.06,3.29	0.029	2.06	1.12,3.79	0.019
**Financial discrimination**								
No stigma	80(31.6)	173(68.4)	Ref					
Stigma	33(19.1)	140(80.9)	0.5	0.35,0.60	0.004	0.49	0.30,0.80	0.004

*Adjusted for age, ethnicity, occupation, religion, parity, education, current family planning current user, age at menarche, first sexual intercourse age

OR- Odds Ratio; **aOR** = adjusted Odds Ratio;

https://doi.org/10.1371/journal.pone.0285771.t003

Policy opposition and avoidance showed no significant association with cervical cancer screening. However, the inference drawn for these domains may not be reliable due to low count (Table 3).

Discussion

Cancer stigma was prevalent among almost a quarter of women residing in suburban central Nepal. However, cancer stigma varied by domain, with the highest endorsement of statements regarding personal responsibility, severity of a cancer diagnosis and financial discrimination, but lower endorsement of statements about awkwardness, avoidance and policy opposition. Women with cancer stigma were 77 percent less likely to have ever been screened compared to those who did not have stigma. Within the domains, awkwardness, severity, and financial discrimination negatively affected screening update, whereas personal responsibility positively affected screening uptake.

One-fourth of the study participants reported ever being screened for cervical cancer of which almost a quarter did not follow the recommended frequency to screen every three to five years, that is three years for pap smear and five years for HPV testing and VIA. Our study findings reported more than three times (26%) the rate of screening uptake compared to a national survey (8%) among women aged 30–49 years [19]. Semi-urban setting, higher women's literacy rate compared to national statistics [21] and regular screening service availability at our study area, may have contributed to a higher cervical cancer screening uptake. However,

the screening coverage is far below the WHO recommended screening target (70 percent) for countries worldwide to achieve by 2030 to get on the path to eliminate cervical cancer [28], and is lower than the national target (50%) for women aged 30–60 years [29].

Our study findings reported quantification of cancer stigma which complements previous qualitative findings from Nepal [30]. Our study exhibits an inverse association between cancer stigma and cervical cancer screening uptake similar to a study conducted in England [14]. Stigma facilitates misconceptions regarding cervical cancer and screening tests and makes women anxious to go for tests. Fear of being diagnosed with cancer and its anticipated implication such as ending a relationship with a partner, family rejection, and loss of livelihood can make women reluctant to take the screening test [31].

Our study revealed that women providing awkwardness statements–anticipated uncomfortable feelings around someone with cancer–are less likely to go for screening. Similar findings were reported from rural Senegal that showed women who had undergone cervical cancer screening are more likely to feel comfortable around someone with cancer [23].

Women providing severity statements–perception of cancer as a terminal disease and never being normal again–are also less likely to receive cervical cancer screening. Such perceptions can make screening seem futile if cancer is believed to be incurable and unpreventable [32, 33].

Participants endorsing financial discrimination statements (i.e. it is acceptable for a bank to refuse loans and mortgage for cancer—related reasons) are less likely to receive cervical cancer screening. Given the substantial financial resources required for cancer treatment [34] and uncertain life expectancy, participants endorsing such sentiments may be reluctant to undergo screening for fear of the financial repercussions.

Interestingly, women endorsing personal responsibility statements such as having cancer is probably their fault demonstrated higher rates of cervical cancer screening uptake. A study from rural Sénégal also showed screened women strongly agree that a diagnosis of cancer is the fault of the person [23]. One possible explanation might be that those who believe that cervical cancer is due to personal responsibility may feel more liable and accountable to seeking out and receiving screening.

To our knowledge, this is the first quantitative study to determine the association between cancer stigma and cervical cancer screening uptake in Nepal. We used CASS—a validated tool with adequate internal validity (Nepali version had Cronbach alpha of 0.81) to measure cancer stigma.

We acknowledge some limitations in our study. First, we could not establish the temporality in the relationship between cancer stigma and screening utilization due to the cross-sectional nature of the study. Second, stigma is a subjective, complex internal feeling that is difficult to comprehensively assess by explicit measurements, and respondents may have adjusted their responses leading to social desirability bias or to mitigate acknowledging their feelings which may under report our findings. Third, there might be chances of selection bias due to recruiting women using convenience sampling techniques which limit generalizability. Fourth, we did not collect and control variables like participants with history of cervical cancer symptoms and signs and a history of cancer in the family which could have confounded the association of cancer stigma with cervical cancer screening uptake. Fifth, cervical cancer screening uptake was self-reported and could have been affected by social desirability or recall bias. Finally, we could not ensure the cervical cancer screening uptake from participants by observing their screening report due to telephonic interview.

Conclusion

Those women who had cancer stigma were less likely to screen for cervical cancer. Particularly, women with stigma related to the subdomains of awkwardness, financial discrimination, and severity were less likely to have received cervical cancer screening, while women reporting personal responsibility stigma were more likely have screening. De-stigmatizing interventions may alleviate cancer stigma and contribute to higher uptake of cervical cancer screening.

Supporting information

S1 Data.
(XLS)

S1 File.
(DOCX)

Acknowledgments

The authors would like to acknowledge all the participants of the study and all those who directly and indirectly helped us throughout our study.

Author Contributions

Conceptualization: Bandana Paneru, Sunila Shakya, Donna Spiegelman, Sangini Seth, Anne Stangl, Archana Shrestha.

Formal analysis: Bandana Paneru, Bikram Adhikari, Archana Shrestha.

Investigation: Bandana Paneru, Aerona Karmacharya, Alina Bharati, Soniya Makaju, Dikshya Kafle.

Methodology: Bandana Paneru, Sunila Shakya, Donna Spiegelman, Sangini Seth, Anne Stangl, Archana Shrestha.

Supervision: Archana Shrestha.

Validation: Archana Shrestha.

Writing – original draft: Bandana Paneru.

Writing – review & editing: Bikram Adhikari, Dikshya Kafle, Sunila Shakya, Donna Spiegelman, Sangini Seth, Anne Stangl, Aamod Dhoj Shrestha, Archana Shrestha.

References

1. Sung H, Ferlay J, Siegel RL, Laversanne M, Soerjomataram I, Jemal A, et al. Global Cancer Statistics 2020: GLOBOCAN Estimates of Incidence and Mortality Worldwide for 36 Cancers in 185 Countries. CA Cancer J Clin. 2021; 71(3): 209–249. https://doi.org/10.3322/caac.21660 PMID: 33538338
2. Arbyn M, Weiderpass E, Bruni L, de Sanjosé S, Saraiya M, Ferlay J, et al. Estimates of incidence and mortality of cervical cancer in 2018: a worldwide analysis. Lancet Glob Heal. 2020; 2(8): 191–203. https://doi.org/10.1016/S2214-109X(19)30482-6 PMID: 31812369
3. Bruni L, Albero G, Serrano B, Mena M, Collado JJ, Gómez D, et al. ICO/IARC Information Center on HPV and Cancer (HPV Information Center). Human Papillomavirus and Related Diseases in the World. Summary Report: 2021. [Date Accessed: 20 Sep 2022].Available: www.hpvcentre.com.
4. Center for Disease Control and Prevention. Cervical Cancer is Preventable | VitalSigns | CDC. [Date Accessed: 15 Aug 2022]. Available: https://www.cdc.gov/vitalsigns/cervical-cancer/index.html.
5. Kim JJ, Burger EA, Regan C, Sy S. Screening for Cervical Cancer in Primary Care: A Decision Analysis for the US Preventive Services Task Force. JAMA. 2018; 320(7): 706–714. https://doi.org/10.1001/jama.2017.19872 PMID: 30140882

6. Ministry of Health and Population. National Guideline for Cervical Cancer Screening and Prevention in Nepal.2010.
7. Link BG, Phelan JC. Conceptualizing stigma. Annu Rev Sociol. 2001; 27: 363–385. doi: 10.1146/ANNUREV.SOC.27.1.363. https://doi.org/10.1080/08870446.2013.834057
8. Shepherd MA, Gerend MA. The blame game: Cervical cancer, knowledge of its link to human papillomavirus and stigma. Psychol Heal. 2014; 29(1): 94–109. https://doi.org/10.1080/08870446.2013.834057 PMID: 24006882
9. Dyer K. 2010 P. K. New Award from Cancer to Sexually Transmitted Infection: Explorations of Social Stigma among Cervical Cancer Survivors. Hum Organ. 2010; 69(4): 321–330. https://doi.org/10.17730/HUMO.69.4.A750670H0784521J
10. Oystacher T, Blasco D, He E, Huang D, Schear R, McGoldrick D, et al. Understanding stigma as a barrier to accessing cancer treatment in South Africa: implications for public health campaigns. Pan Afr Med J. 2018; 29: 1–12. https://doi.org/10.4314/pamj.v29i1
11. O'Connor M, O'Leary E, Waller J, Gallagher P, Martin CM, O'Leary JJ, et al. Socio-economic variations in anticipated adverse reactions to testing HPV positive: Implications for the introduction of primary HPV-based cervical screening. Prev Med (Baltim). 2018; 115: 90–96. https://doi.org/10.1016/j.ypmed.2018.08.017 PMID: 30144488
12. Gupta A, Dhillon PK, Govil J, Bumb D, Dey S, Krishnan S. Multiple Stakeholder Perspectives on Cancer Stigma in North India. Asian Pac J Cancer Prev. 2015; 16: 6141–6147. https://doi.org/10.7314/apjcp.2015.16.14.6141 PMID: 26320509
13. Weiss MG, Ramakrishna J. Stigma interventions and research for international health. Lancet. Elsevier B.V.; 2006. pp. 536–538. https://doi.org/10.1016/S0140-6736(06)68189-0 PMID: 16473134
14. Nyblade L, Stockton M, Travasso S, Krishnan S. A qualitative exploration of cervical and breast cancer stigma in Karnataka, India. BMC Womens Health. 2017; 17(58): 1–15. https://doi.org/10.1186/s12905-017-0407-x PMID: 28768506
15. Vrinten C, Gallagher A, Waller J, Marlow LAV, 'Vrinten C, Gallagher A, et al. Cancer stigma and cancer screening attendance: A population based survey in England. BMC Cancer. 2019; 19(566). https://doi.org/10.1186/s12885-019-5787-x PMID: 31185949
16. Racey CS, Gesink DC. Barriers and Facilitators to Cervical Cancer Screening Among Women in Rural Ontario, Canada: The Role of Self-Collected HPV Testing. J Rural Heal. 2016; 32(2): 136–145. https://doi.org/10.1111/jrh.12136 PMID: 26265118
17. Buchanan Lunsford N, Ragan K, Smith JL, Saraiya M, Aketch M. Environmental and Psychosocial Barriers to and Benefits of Cervical Cancer Screening in Kenya. National Library of Medicine.2017; 22(2):173–181. https://doi.org/10.1634/theoncologist.2016-0213 PMID: 28167567
18. Gele AA, Qureshi SA, Kour P, Kumar B, Diaz E. Barriers and facilitators to cervical cancer screening among Pakistani and Somali immigrant women in Oslo: a qualitative study. Int J Womens Health. 2017; 9: 487. https://doi.org/10.2147/IJWH.S139160 PMID: 28740435
19. Nepal STEPS Survey 2019 Fact Sheet. [Date accessed: 26 Aug 2021]. Available: https://drive.google.com/file/d/1cwnheqGQKmtguhRtLiNrhZuca0_SXP1d/view.
20. Greibe Andersen J, Shrestha AD, Gyawali B, Neupane D, Kallestrup P. Barriers and facilitators to cervical cancer screening uptake among women in Nepal–a qualitative study. Women Heal. 2020; 392:963–974. https://doi.org/10.1080/03630242.2020.1781742 PMID: 32643576
21. Nepal Census 2011 District Profiles (Demography)—Humanitarian Data Exchange. [Date accessed: 17 Aug 2021]. Available: https://data.humdata.org/dataset/nepal-census-2011-district-profiles-demography.
22. Wang H, Chow S-C. Sample Size Calculation for Comparing Proportions. Wiley Encyclopedia of Clinical Trials. Hoboken, NJ, USA: John Wiley & Sons, Inc.; 2007. https://doi.org/10.1002/9780471462422.eoct005
23. Ongtengco N, Thiam H, Collins Z, de Jesus E Lou, Peterson CE, Wang T, et al. Role of gender in perspectives of discrimination, stigma, and attitudes relative to cervical cancer in rural Sénégal. PLoS One. 2020; 15(4): e0232291. https://doi.org/10.1371/journal.pone.0232291 PMID: 32343755
24. Dhimal M, Bista B, Bhattarai S, Hyder MKA, Agarwal N, Rani M JA. Noncommunicable Disease Risk Factors: STEPS Survey Nepal 2019. Nepal Health Res Counc. 2019; 284. Available: https://www.who.int/docs/default-source/nepal-documents/ncds/ncd-steps-survey-2019-compressed.pdf?sfvrsn=807bc4c6_2.
25. Ministry of Health. Nepal Demographic and Health Survey 2016. Kathmandu Nepal Minist Heal Nepal. 2017.
26. Marlow LAV, Wardle J. Development of a scale to assess cancer stigma in the non-patient population. BMC Cancer. 2014; 14: 285. https://doi.org/10.1186/1471-2407-14-285 PMID: 24758482

27. Binomial Proportion Confidence Interval—Clopper-Pearson Interval. [Date accessed: 2 Jan 2022]. Available:https://www.liquisearch.com/binomial_proportion_confidence_interval/clopper-pearson_interval

28. New recommendations for screening and treatment to prevent cervical cancer. [Date accessed: 11 Apr 2022]. Available:https://www.who.int/news/item/06-07-2021-new-recommendations-for-screening-and-treatment-to-prevent-cervical-cancer

29. Cervical and Breast Cancer Screening Program Implementation Guideline 2077—Public Health Update. [Date accessed: 29 Nov 2021]. Available: https://publichealthupdate.com/cervical-and-breast-cancer-screening-program-implementation-guideline-2077/

30. Shrestha G, Mulmi R, Phuyal P, Thakur RK, Siwakoti B. Experiences of cervical cancer survivors in Chitwan, Nepal: A qualitative study. Pradhan PMS, editor. PLoS One. 2020; 15: e0234834. https://doi.org/10.1371/journal.pone.0234834 PMID: 33151965

31. Rosser JI, Njoroge B, Huchko MJ. Cervical cancer stigma in rural Kenya: What does HIV have to do with it? J Cancer Educ. 2016; 31: 413. https://doi.org/10.1007/s13187-015-0843-y PMID: 25982550

32. White HL, Mulambia C, Sinkala M, Mwanahamuntu MH, Parham GP, Moneyham L, et al. 'Worse than HIV' or 'not as serious as other diseases'? Conceptualization of cervical cancer among newly screened women in Zambia. Soc Sci Med. 2012; 74: 1486–1493. https://doi.org/10.1016/j.socscimed.2012.01.028 PMID: 22459188

33. Olsson E, Lau M, Lifvergren S, Chakhunashvili A. Community collaboration to increase foreign-born women´s participation in a cervical cancer screening program in Sweden: a quality improvement project. Int J Equity Heal 2014 131. 2014; 13: 1–10. https://doi.org/10.1186/S12939-014-0062-X PMID: 25106490

34. Subramanian S, Trogdon J, Ekwueme DU, Gardner JG, Whitmire JT, Rao C. Cost of cervical cancer treatment: implications for providing coverage to low-income women under the Medicaid expansion for cancer care. Womens Health Issues. 2010; 20: 400–405. https://doi.org/10.1016/j.whi.2010.07.002 PMID: 21050999
