# Supplementary material for: Socio-economic factors associated with cancer stigma among apparently healthy women in two selected municipalities Nepal
Source: PLoS One. 2024 Dec 16;19(12):e0301059. doi: 10.1371/journal.pone.0301059 (PMC11649127; doi:10.1371/journal.pone.0301059)
Supplement: Attachment — Submitted filename: Response to Reviewers.docx [file pone.0301059.s004.docx]

**Jul 19, 2024**

Dear Reviewers,

Thank you very much for your valuable comments . They were very helpful in improving this manuscript. I have incorporated the suggested changes into the manuscript to the best of my ability. Please find the response to the comments.

**Reviewer 1**

Title

1. Title says semi-urban Nepal which miss lead authors because it seems to be carried out in two purposively selected municipalities of Nepal and this result is not generalizable to all semi-urban area as you have mentioned in the limitation section of the manuscript.

**Response:** Thank you very much for your response. We will revise the title as follow; “**Socio-Economic Factors Associated with Cancer Stigma among Apparently Healthy Women in two selected municipalities Nepal**

**Methods**

2. Please check if the regression analysis is multivariable or multivariate ?

**Response: That was an error. Thank you for noticing. We have revised the term** multivariate to multivariable**;**

We performed multivariable linear regression analysis to determine the socio-demographic factors associated with cancer stigma, selecting the variables in our model based on literature review.

3. Please clarify how variables were selected for multivariable regression analysis.

**Response**: Variables were selected for multivariable regression analysis based on previous literatures and prior knowledge. We have added this information in the methods section

ADD THE INFO

4. Have you performed the diagnostics to test for the assumptions of the linear regression analysis. If yes, please write in details about how you performed the diagnostics. In addition, How was the distribution of the residuals?

Have you checked for the multicollinearity ?

**Response**: Yes, we performed the diagnostics for linear regression. We checked for linearity, independence, normality, and equal variances of the residuals; and collinearity We have added this information in the method section as follows.

We checked the assumptions of linearity, independence, normality, and equal variance for linear regression in our analysis. We used a scatterplot of residuals versus predicted values to assess the linearity and equal variance assumptions. To check for normality, we examined a histogram and a normal probability plot of the residuals. We used the GEE model to address clustering (dependence) by ward, which has been addressed in the methods.Additionally, to detect multicollinearity, we used the variance inflation factors (VIF) method. The mean VIF was around 2.10, indicating that multicollinearity was not problematic.

5. You have included SD in the results section. Please write its full form in the data analysis section and the bottom of the respective tables in which they are used.

**Response:**Thank you very much for your comments. We have added the full form of SD as Standard Deviation in data analysis section and the bottom of the respective tables

**6. Consider writing SD in uniform way throughout the manuscript [abstract and text part used ± whereas line 170 and table 1 used mean(SD)]**

**Response:** Thank you very much.We have made **mean(SD)** as in a uniform way throughout the manuscript.

**7. variables like at least one CCS screening can be a confounder. Why it is not adjusted in multivariable regression**

**Response:** We didn't adjust for screening uptake because it could serve as a mediator in the relationship between independent variables and cancer stigma. For example, for age, older individuals might have higher screening rates due to more frequent healthcare interactions, which could reduce stigma through increased awareness and education. Adjusting for this mediator could lead to over-adjustment bias**.**[[1]](https://paperpile.com/c/APlT2j/fCT0)

**8. There are chances of clustering of data, community of people sharing common characteristics. it is better to address the clustering of the data.**

**Response:**Yes, will utilize Generalized Estimating Equations (GEE) with multivariate linear regression, exchangeable working correlation, and robust variance to account for the clustering effect of community people on stigma scores, using the ward level as the clustering level. However, the results from the GEE analysis were similar to those obtained from the multivariable linear regression. The revised table will be as follows;

| Characteristics | Univariable | | Multivariable | |
| --- | --- | --- | --- | --- |
|  | Difference in CASS score (95% CI) | p-value | Difference in CASS score  (95% CI) | p-value |
| *Age (years), Mean (SD) | 0.2 (0.10, 0.30) | 0.002 | 0.11 (0.02, 0.20) | 0.01 |
| Education (years of formal education), Mean (SD) | 0.04 (-0.05, -0.02) | <0.001 | -0.02 (-0.03, -0.01) | <0.001 |
| Ethnicity |  | | | |
| Brahmin/Chhetri | Ref | 0.67 |  | 0.31 |
| Damai/Sarki/Gaaine/Baadi | 0.03 (-0.39, 0.40) |  | -0.07 (-0.34, 0.24) |  |
| Magar/Tamang/Rai/Limbu | 0.19 (-0.70, 0.37) |  | -0.23 (- 0.49, 0.04) |  |
| Newar | -0.08 (-0.29, 0.12) |  | 0.04 (-0.03, 0.12) |  |
| Sherpa/Bhote | 0.09 (-0.31, 0.50) |  | 0.07 (-0.28, 0.43) |  |
| Occupation |  | | | |
| Business | Ref | 0.53 |  | 0.49 |
| Farmer | 0.37(0.08, 0.67) |  | 0.01 (-0.09, 0.10) |  |
| Home-maker | 0.02 (-0.29, 0.33) |  | -0.06 (-0.23, 0.12) |  |
| Others | 0.15 (-0.18, 0.48) |  | 0.04 (-0.14, 0.23) |  |
| Unemployed | 0.59 (-0.20, 1.38) |  | 0.07 (-0.27, 0.41) |  |
| Parity, Mean (SD) | 0.09 (-0.01, 0.18) |  | 0.10 ( -0.24, 0.46) | 0.98 |
| Religion |  |  |  |  |
| Buddhist | Ref | 0.04 |  | 0.22 |
| Christian | -0.02 (-0.57, 0.63) |  | -0.13 (-0.63, 0.36) |  |
| Hindu | -0.19 (-0.59, 0.19) |  | -0.16 (-0.40, 0.07) |  |

**Results:**

**9. Line 197 CI can be opened in line 165**

**consider writing p-value in uniform way throughout the paper [for example: there is different in line 202 and line 198 ]**

**Response:** Thank you very much for your comments. We will write p-value in a uniform way throughout the paper .

**10. Reference 31 seems to be the first paper linked to this paper. The sample size is 426. Table 1 from published paper(**[**https://journals.plos.org/plosone/article?id=10.1371/journal.pone.0285771**](https://journals.plos.org/plosone/article?id=10.1371/journal.pone.0285771)**) doesn't match with the table 1 of this paper. For example: there were 184 brahmin/chhetri/sanyashi/thakuri but later it is 182. similarly there are slight differences between two papers. I think the analysis has to be redo after correcting the data .**

**Response:** This was an error. We will rectify this.

**11. Clarify what other occupations or ethnicity includes in methods or at the bottom of the tables.**

**Response:** We have clarified other occupations at the bottom of the tables. Other occupations include cleaner, helper, tailor, labor, parlor,and teacher.

**12. Readers would like to read the crude CASS score for each categories of selected variables along with the overall CASS score**

**Response:** We have added the crude CASS score for each domain and over all CASS score with the category of the selected variables.

We will add table 3 in the result section as follows;

Table 3: Cancer stigma scale (CASS) mean score in each domain for selected variable (n=426)

| Characteristics | Overall score | Awkwardness | Severity | Avoidance | Policy Opposition | Personal Responsibility | Financial discrimination |
| --- | --- | --- | --- | --- | --- | --- | --- |
| **Age (years)** |  |  |  |  |  |  |  |
| less than 40 | 2.4(0.6) | 2.3(1.3) | 2.9(1.3) | 1.5(0.7) | 1.5(0.7) | 3.8(1.3) | 2.7(1.5) |
| 40 to 49 | 2.6(0.6) | 2.5(1.3) | 3.3(1.2) | 1.7(0.9) | 1.3(0.6) | 3.9(1.3) | 3.3(1.7) |
| 50 and above | 2.7(0.5) | 2.5(1.2) | 3.5(1.2) | 1.9(1.1) | 1.3(0.5) | 4.0(1.1) | 2.9(1.5) |
| ***Education** |  |  |  |  |  |  |  |
| No formal education | 2.8(0.7) | 2.6(1.3) | 3.5(1.3) | 1.9(1.1) | 1.4(0.6) | 4.2(1.2) | 2.9(1.6) |
| Primary | 2.6(0.6) | 2.6(1.2) | 3.5(1.1) | 1.7(1.0) | 1.3(0.4) | 3.9(1.3) | 2.7(1.5) |
| Secondary | 2.5(0.6) | 2.3(1.3) | 3.0(1.1) | 1.5(0.7) | 1.2(0.5) | 3.7(1.3) | 3.0(1.5) |
| Above secondary | 2.4(0.5) | 2.3(1.1) | 2.9(1.2) | 1.5(0.7) | 1.3(0.6) | 3.7(1.2) | 3.0(1.7) |
| **Ethnicity** |  | | | |  |  |  |
| Brahmin/Chhetri | 2.6(0.6) | 2.4(1.2) | 3.2(1.3) | 1.7 (1.0) | 1.4(0.6) | 4.0(1.1) | 2.7(1.5) |
| Damai/Sarki/Gaaine/Baadi | 2.5(0.5) | 2.1(1.1) | 3.2(1.)2 | 1.6(0.8) | 1.3(0.4) | 4.7(1.2) | 2.1(1.4) |
| Magar/Tamang/Rai/Limbu | 2.2(0.6) | 2.2(1.0) | 2.5(1.4) | 1.6(0.7) | 1.2(0.3) | 4.2(1.1) | 1.7(1.1) |
| Newar | 2.6(0.6) | 2.5(1.3) | 3.2(1.2) | 1.6(0.8) | 1.3(0.5) | 3.5(1.4) | 3.4(1.6) |
| Sherpa/Bhote | 2.8(0.7) | 2.5(1.5) | 3.6 (1.4) | 1.7(0.9) | 1.3(0.5) | 4.5(1.0) | 2.9(1.8) |
| **Occupation** |  | | | |  |  |  |
| Business | 2.6(0.6) | 2.4(1.4) | 3.1(1.2) | 1.5(0.7) | 1.3(0.5) | 3.8(1.4) | 3.2(1.9) |
| Farmer | 2.7(0.6) | 2.4(1.3) | 3.3(1.3) | 1.7(0.9) | 1.3(0.6) | 4.1(1.2) | 3.0(1.7) |
| Home-maker | 2.5(0.6) | 2.3(1.3) | 3.0(1.3) | 1.7(0.9) | 1.3(0.5) | 3.8(1.3) | 2.8(1.4) |
| Others | 2.6(0.7) | 2.5(1.3) | 3.0(1.2) | 1.6(1.0) | 1.4(0.7) | 3.7(1.3) | 2.8(1.4) |
| Unemployed | 2.6(0.4) | 2.7(1.0) | 2.9(1.3) | 1.4(0.7) | 1.7(0.8) | 3.8(1.5) | 3.1(1.2) |
| **Parity** |  |  |  |  |  |  |  |
| Zero | 2.1(0.7) | 1.9(0.7) | 2.1(0.7) | 1.5(0.7) | 1.0(0.3) | 4.1(1.4) | 2.2(1.9) |
| One to two | 2.6(0.6) | 2.4(1.3) | 3.1(1.3) | 1.6(0.8) | 1.3(0.6) | 3.9(1.3) | 2.9(1.6) |
| More than two | 2.7(0.6) | 2.5(1.3) | 3.4(1.3) | 1.8(0.7) | 1.3(0.5) | 3.9(1.)2 | 3.0(1.6) |
| **Religion** |  |  |  |  |  |  |  |
| Buddhist | 2.8(0.7) | 2.6(1.4) | 3.7(1.2) | 1.7(0.8) | 1.3(0.5) | 4.4(1.1) | 3.2(1.9) |
| Christian | 2.6(0.7) | 2.1(1.2) | 3.1(1.2) | 1.6(0.9) | 1.5(0.9) | 4.8(0.9) | 2.4(1.7) |
| Hindu | 2.6(0.6) | 2.4(1.3) | 3.2(1.3) | 1.7(0.9) | 1.3(0.6) | 3.8(1.3) | 3.0(1.6) |

*Education(years of formal education completed) 0=No formal education ; 1/5= primary education; 6/10=secondary education; and 11/max=above secondary

Description of table 3 is written in revised manuscript

**Discussion:**

**13. Line 212 second part to line 217 is confusing. I think you are summarizing the result but abruptly explanation of the domains comes.**

**Response**: We have updated this section and summarize the main findings in the first paragraphs as follows:

Our study found a low level of general cancer stigma among apparently healthy women in semi-urban Nepal. However, we observed high levels of cancer stigma in the domains of personal responsibility, severity, and financial discrimination. The cancer stigma score was lowest in the policy opposition domain. Cancer stigma scores were higher among older individuals and those with lower levels of formal education.

**14. Line 226 -line 230: You have been comparing CASS score between Nepal and UK and abruptly you are saying that stigma varies across diseases... This is irrelevant in this paragraph.**

**15. You are comparing with High income countries. It would be better if it is possible to compare with the low and middle income countries.**

Response: Thank you very much for your feedback. There are very limited studies on cancer stigma in low- and middle-income countries. We have included literature from India and Nepal in the discussion section.

We have revised this paragraph as follows and incorporated the study from Nepal and India . Discussion is revised as follows;

In second paragraph

In previous studies exploring cancer stigma, consistently low levels of stigma have been reported, though these levels vary across different domains. Similar to our findings, a recent community-based study conducted in India[[2]](https://paperpile.com/c/APlT2j/bpiV) and a hospital-based study in Nepal [[3]](https://paperpile.com/c/APlT2j/E4nK) reported high stigma in the domains of severity, awkwardness, and personal responsibility, with the lowest levels in policy opposition. Additionally, a cross-sectional study conducted in the UK showed higher cancer stigma in the domains of personal responsibility and severity, similar to our study. [[4]](https://paperpile.com/c/APlT2j/qlMCL) In contrast to women from the UK, our study population exhibited a higher total mean cancer stigma score across five sub-domains: severity, awkwardness, financial discrimination, personal responsibility, and avoidance. However, our study participants demonstrated a lower mean stigma score in the domain of policy opposition compared to English women, indicating less support for government funding toward cancer care and treatment.[[4,5]](https://paperpile.com/c/APlT2j/qlMCL+90hAF)

**16. Line 234: What exact difference in health financing and community structure that resulted in low stigma score in policy opposition. Is there any specific study that reported that difference in health financing and community structure affecting stigma score.**

**Response:** We have added the explanation in the discussion section with citation

**17. Line 265: multivariate or multivariable??**

**Response:** Has been addressed in number 2. Multivariate is revised to multivariable throughout the paper.

**References
18. Reference 29 is not valid. Need to cite the original source.**

**Response:** revised citation as follow;

[Ministry of Health and Population. Female Community Health Programme [Internet]. [cited 2024 Jul 11]. Available from:](http://paperpile.com/b/LUUwQX/Dybs)<https://mohp.gov.np/program/female-community-health-programme/en>

**19. Reference 41 is to be corrected**

**Response:** Rectified as follow;

[Acharya U. Nepal Social Media Users Survey 2021. 2022 May; Available from:](http://paperpile.com/b/LUUwQX/xH6bU) <https://research.butmedia.org/wp-content/uploads/2022/06/SocialMediaSurvey_Nepal_2021_CMR.pdf>

**20. Reference 28 to be corrected**

**Response:** Rectified as follow;

[Brooks S. Comparing Two Proportions – Sample Size [Internet]. [cited 2022 Jul 31]. Available from:](http://paperpile.com/b/LUUwQX/2RW2E) <https://select-statistics.co.uk/calculators/sample-size-calculator-two-proportions/>

**Reviewer 2**

**While reviewing the paper, I came across two other similar papers published from the same data set by the same authors.
(1) Shrestha A, Stangl AL, Paneru B, Poudel L, Karmacharya A, Makaju S, et al. Validation of
361 the Cancer Stigma Scale in Nepalese Women. Asian Pac J Cancer Prev. 2023 Jan
362 1;24(1):207–14.
(2) Paneru B, Karmacharya A, Bharati A, Makaju S, Adhikari B, Kafle D, et al. (2023) Association between cancer stigma and cervical cancer screening uptake among women of Dhulikhel and Banepa, Nepal. PLoS ONE 18(5): e0285771.** [**https:/**](https://doi.org/10.1371/journal.pone.0285771)

**The first paper presents the validity of cancer stigma scale in Nepalese context while the second paper measures the prevalence of cancer stigma and its association with cancer screening uptake. This manuscript also measures the prevalence of cancer stigma, and further identify its associated socio-demographic factors. Authors have not cited the second paper which looks very similar to this manuscript. The prevalence of cancer stigma is presented in both papers; the only difference is that the figures are reported in either percentage or mean. The socio-demographic characteristics of the participants (Table 1) are presented in all the three papers which is same and hence redundant. The authors could have mentioned that the findings are reported in previous work and hence avoid redundant information in the manuscript.
The authors must provide a sound scientific rationale for the submitted work and clearly reference and discuss the existing literature. The authors need to provide adequate justification for the novelty of their work.**

**Response:** Thank you very much for your comments. Our team did two cancer stigma studies in the same study population.The first paper focused on the validity of the tool in the Nepalese setting. The second paper aims to assess the association between cancer stigma and screening uptake, adjusting for other socio-demographic variables. The current paper aims to determine the cancer stigma score for each item, each domain, and the overall score. The novelty of this study lies in its focus on quantitatively measuring cancer stigma within the non-cancer population, a topic that has been sparsely explored in previous research. Additionally, prior studies have not provided detailed assessments of stigma associated with individual items and domains. This information will be valuable in designing targeted stigma reduction interventions by identifying specific areas that need attention.

This is added in the introduction section in the revised manuscript.

**Minor comments
Abstract**

1. **background: Mention it as either general population or general female population in Nepal**

**Response:** This is the general female population. We have updated this in our background.

2. **results: could you present the measurement scale as well? You can present the scale in methods. This will help to understand the mean score in practical terms**

**Response:** Thank You very much for your comments. We have explained briefly about it in the method section.

**conclusion: the sentence is not clear. Better to rephrase as 'While..., some subdomains of stigma were higher among ---in Nepal'**

**Response:** Conclusion is rephrased as follows;

While overall cancer stigma was low, some domains of stigma were higher among women in a suburban area in central Nepal; thus, indicating that cancer stigma persists in this region despite its low overall prevalence.

**Introduction:**

**the year 2020 is redundant (1st paragraph)**

**Response:** Revised 1st paragraph removing 2020 repeatedly.

**who does the non-patient refer to? A more clarity is required. (3rd paragraph)**

**Response:**

Revised third paragraph as follows;Although people often stigmatized cancer, there is a limited exploration of public stigma in healthy general female populations.

**Revise first paragraph as follows;**Cancer is the leading cause of death worldwide, with age-standardized mortality of 100 per 100,000 population in 2020.[[9]](https://paperpile.com/c/APlT2j/1mrY5) The majority of cancer deaths occur in low- and middle-income countries.[[9]](https://paperpile.com/c/APlT2j/1mrY5) In Nepal, the age-standardized cancer incidence rate is 80 per 100,000 with a mortality rate of 54 per 100,000.[[9]](https://paperpile.com/c/APlT2j/1mrY5) Despite recent improvements in treatment and survival, cancer is still a stigmatized disease,[[10–12]](https://paperpile.com/c/APlT2j/rUKTU+rlSsF+Q1L7u) and one of the most feared illnesses.[[13]](https://paperpile.com/c/APlT2j/Fnn7M)

**Introduction: Could you mention the full form of CASS initially? You can abbreviate afterwards (last paragraph)**

**Response:** Thank You very much for your comments. We have now provided the full form of CASS initially as Cancer Stigma Scale and abbreviated afterwards.

**Method: better to present how many items were under each of the six domains (Measures)**

**Response:** Thank You very much. We will add the number of items under six domains in the method section. Revised measures as follows;

The CASS consists of 25 items that assess six domains: (a) awkwardness-5 items, which measures how comfortable people feel around someone with cancer; (b) severity-5 items, which evaluates the expected severity of cancer consequences and the likelihood of recovery; (c) avoidance-5 items, which assesses the extent to which people avoid cancer patients and maintain physical distance from them; (d) personal responsibility-4 items, which determines how much a person's actions contribute to their cancer; (e) policy opposition-3 items, which gauges the perceived responsibility of the government and the public in the care and treatment of cancer patients; and (f) financial discrimination-3 items, which measures the anticipated deprivation of benefits to cancer patients from banking and insurance services

**Results: How was age treated in the linear regression model? could you make it more clear by taking readers' attention?**

**Response:**Age was treated as a continuous variable in the regression model. We have revised the interpretation as follows to make it more clear.

There is a positive association between age and cancer stigma score. When comparing two groups with a ten-year age difference, the older group had a higher cancer stigma score of 0.18 units compared to the younger group, after accounting for differences in education, ethnicity, occupation, number of children, and religion (95% CI: 0.04-0.20; p-value=0.04).

**Discussion: what is your notion of categorizing higher and lower levels of cancer stigma when you are presenting means of the sub-domain? (1st paragraph)**

Response: We have revised the term higher as high and lower as low in 1st paragraph of discussion

**Discussion: what about other mass media platforms? the sources of information regarding cancer may be varied in Nepalese context (5th paragraph)**

Response: We have now Added other platforms as well in the fifth paragraph as follows;

Other mass media in Nepal includes radio, television but could not find the literature regarding its use among women in Nepal. In the context of Nepal one common source of information on cancer or any health issues is Female Community Health Volunteers in their Health Mother Group meetings and 35 to 45 years old women were more likely to attend these meetings compared to older groups.[[14]](https://paperpile.com/c/APlT2j/KDDh)

We have added these information and revise

**Conclusion: The conclusion that prevalence of cancer is low needs further explanation while referring to the mean scores.**

Response: Adding the mean scores, the conclusion regarding the prevalence of low cancer stigma is revised as follows:

In conclusion, this study found that overall cancer stigma was low, with a mean stigma score of 2.6 (0.6), indicating its presence among women in a suburban area in central Nepal.

**Proper use of abbreviation is recommended throughout the paper.**

Response: Thank You very much for your feedback. We will ensure proper use of abbreviation throughout the paper.

**References**

1. [Lu H, Cole SR, Platt RW, Schisterman EF. Revisiting Overadjustment Bias. Epidemiology. 2021;32: e22–e23.](http://paperpile.com/b/APlT2j/fCT0)

2.	[Mohana Priya S, Annie ET. Cancer stigma among the caregivers of cancer patients, a cross-sectional study from a secondary care hospital in Andhra Pradesh, South India. International Journal of Science and Research Archive. 2024;12: 2097–2102.](http://paperpile.com/b/APlT2j/bpiV)

3.	[Shrestha R, Shrestha G, Paneru P. Cancer Stigma in Non-patient Population Visiting B&B Hospital, Lalitpur, Nepal: A Descriptive Cross-sectional Study. Asian Pacific Journal of Cancer Care. 2023;8: 275–279.](http://paperpile.com/b/APlT2j/E4nK)

4.	[Vrinten C, Gallagher A, Waller J, Marlow LAV. Cancer stigma and cancer screening attendance: a population based survey in England. BMC Cancer. 2019;19: 566.](http://paperpile.com/b/APlT2j/qlMCL)

5.	[Marlow LAV, Wardle J. Development of a scale to assess cancer stigma in the non-patient population. BMC Cancer. 2014;14: 285.](http://paperpile.com/b/APlT2j/90hAF)

6.	[Jones N, Campion B, Keeling M, Greenberg N. Cohesion, leadership, mental health stigmatisation and perceived barriers to care in UK military personnel. J Ment Health. 2018;27: 10–18.](http://paperpile.com/b/APlT2j/q2ly)

7.	[Link, B.G. and Phelan, J.C. (2001) Conceptualizing stigma. Annual Review Sociology, 27, 363-385. - References - Scientific Research Publishing. [cited 31 Jul 2022]. Available:](http://paperpile.com/b/APlT2j/CsPJ) <https://www.scirp.org/reference/ReferencesPapers.aspx?ReferenceID=950639>

8.	[Hatzenbuehler ML, Bellatorre A, Lee Y, Finch BK, Muennig P, Fiscella K. Structural stigma and all-cause mortality in sexual minority populations. Soc Sci Med. 2014;103: 33–41.](http://paperpile.com/b/APlT2j/PRAx)

9.	[Global cancer data by country. In: WCRF International [Internet]. 28 Mar 2022 [cited 11 Jul 2023]. Available:](http://paperpile.com/b/APlT2j/1mrY5) <https://www.wcrf.org/cancer-trends/global-cancer-data-by-country/>

10.	[Holland JC, Kelly BJ, Weinberger MI. Why psychosocial care is difficult to integrate into routine cancer care: stigma is the elephant in the room. J Natl Compr Canc Netw. 2010;8: 362–366.](http://paperpile.com/b/APlT2j/rUKTU)

11.	[Peters-Golden H. Breast cancer: varied perceptions of social support in the illness experience. Soc Sci Med. 1982;16: 483–491.](http://paperpile.com/b/APlT2j/rlSsF)

12.	[Phelan SM, Griffin JM, Jackson GL, Zafar SY, Hellerstedt W, Stahre M, et al. Stigma, perceived blame, self-blame, and depressive symptoms in men with colorectal cancer. Psychooncology. 2013;22: 65–73.](http://paperpile.com/b/APlT2j/Q1L7u)

13.	[Else-Quest NM, Jackson TL, Corrigan P. The Stigma of Disease and Disability. 2014.](http://paperpile.com/b/APlT2j/Fnn7M)

14.	[Acharya A, Chang C-L, Chen M, Weissman A. Facilitators and barriers to participation in health mothers’ groups in improving maternal and child health and nutrition in Nepal : A mixed-methods study. BMC Public Health. 2022;22: 1660.](http://paperpile.com/b/APlT2j/KDDh)
