# Supplementary material for: Socio-economic factors associated with cancer stigma among apparently healthy women in two selected municipalities Nepal
Source: PLoS One. 2024 Dec 16;19(12):e0301059. doi: 10.1371/journal.pone.0301059 (PMC11649127; doi:10.1371/journal.pone.0301059)
Supplement: Attachment — Submitted filename: Response to Reviewers-2 .docx [file pone.0301059.s005.docx]

November 14, 2024

Dear Reviewers,

Thank you very much for your valuable comments . They were very helpful in improving this manuscript. I have incorporated the suggested changes into the manuscript to the best of my ability. Please find the response to the comments.

1. However, In the introduction section, justification of the need of the study should be adequately explained as commented by reviewer 2. For instance, what were the findings related to cancer stigma in your previous work and what would you add in your current research? Please add in the introduction section to justify. In addition, on which basis the authors had chosen the factors (socio-economic) associated with stigma among the many factors? As this study aims to find out the factors associated with stigma it is important to justify the selected variables.

**Response:** We will add an introduction section as follows;

Our team previously conducted two studies on cancer stigma. The first assessed the validity of the Cancer Stigma Scale (CASS) in the Nepalese context, and the second examined the relationship between cancer stigma and cervical cancer screening uptake, finding that women with higher stigma were less likely to participate in screening. To better understand this, it is important to examine the level and distribution of stigma, identify the domains where stigma is most prevalent, and analyze which socio-economic groups are more affected. Therefore,in this study, we aim to determine item-specific, domain-specific, and overall cancer stigma scores, as well as assess the socio-economic factors associated with cancer stigma. Given the limited research on socio-economic influences on cancer stigma, we selected these factors based on a review of prior literature and our professional judgment.

2. Line 96-100 needs to rewrite/rephrase to make it more clearer and academic

**Response:** We will revise it as follows;

Our team previously conducted two studies on cancer stigma. The first assessed the validity of the Cancer Stigma Scale (CASS) in the Nepalese context, and the second examined the relationship between cancer stigma and cervical cancer screening uptake. In this study, we aim to determine item-specific, domain-specific, and overall cancer stigma scores, as well as assess the socio-economic factors associated with cancer stigma.

Note: In the revised manuscript it is incorporated with comment 1.

3. Could you make it more clearer again about table 3 regarding age? What do you mean by 10 years difference? It is suggested that you mention details in the data analysis section.

**Response:**Thank you very much for your feedback. We will add it as data analysis as follows;

For age variables, we presented coefficients in 10-year increments to enhance interpretability, as one-year changes yield small, less meaningful coefficients, while 10-year groupings capture more substantial age-related trends.

4. Conclusion should be made in a separate section and it should reflect the title and aim of your study. Consider rewriting the conclusion section.

**Response:** We will separate conclusion and revise it as follows;

**Conclusion**: In conclusion, this study found that overall cancer stigma was low, with a mean stigma score of 2.6 (SD = 0.6), indicating its presence among women in a suburban area of central Nepal. Cancer stigma was highest in the domains of personal responsibility, severity, and financial discrimination.Stigma was higher among older women and lower among those with higher education. Since stigma may affect participation in cancer screening, stigma-reduction interventions targeting older and less educated women are recommended.
